# Regulation of gene expression in ovarian cancer cells by luteinizing hormone receptor expression and activation

**DOI:** 10.1186/1471-2407-11-280

**Published:** 2011-06-28

**Authors:** Juan Cui, Brooke M Miner, Joanna B Eldredge, Susanne W Warrenfeltz, Phuongan Dam, Ying Xu, David Puett

**Affiliations:** 1 Department of Biochemistry and Molecular Biology, University of Georgia, Athens, GA 30602, USA; 2 Institute of Bioinformatics, University of Georgia, Athens, GA 30602, USA; 3 College of Computer Science and Technology, Jilin University, Changchun, Jilin, 130012, China

**Keywords:** Ovarian cancer, gonadotropin, luteinizing hormone, luteinizing hormone receptor, SKOV3 cells, microarray

## Abstract

**Background:**

Since a substantial percentage of ovarian cancers express gonadotropin receptors and are responsive to the relatively high concentrations of pituitary gonadotropins during the postmenopausal years, it has been suggested that receptor activation may contribute to the etiology and/or progression of the neoplasm. The goal of the present study was to develop a cell model to determine the impact of luteinizing hormone (LH) receptor (LHR) expression and LH-mediated LHR activation on gene expression and thus obtain insights into the mechanism of gonadotropin action on ovarian surface epithelial (OSE) carcinoma cells.

**Methods:**

The human ovarian cancer cell line, SKOV-3, was stably transfected to express functional LHR and incubated with LH for various periods of time (0-20 hours). Transcriptomic profiling was performed on these cells to identify LHR expression/activation-dependent changes in gene expression levels and pathways by microarray and qRT-PCR analyses.

**Results:**

Through comparative analysis on the LHR-transfected SKOV-3 cells exposed to LH, we observed the differential expression of 1,783 genes in response to LH treatment, among which five significant families were enriched, including those of growth factors, translation regulators, transporters, G-protein coupled receptors, and ligand-dependent nuclear receptors. The most highly induced early and intermediate responses were found to occupy a network impacting transcriptional regulation, cell growth, apoptosis, and multiple signaling transductions, giving indications of LH-induced apoptosis and cell growth inhibition through the significant changes in, for example, tumor necrosis factor, Jun and many others, supportive of the observed cell growth reduction in *in vitro *assays. However, other observations, e.g. the substantial up-regulation of the genes encoding the endothelin-1 subtype A receptor, stromal cell-derived factor 1, and insulin-like growth factor II, all of which are potential therapeutic targets, may reflect a positive mediation of ovarian cancer growth.

**Conclusion:**

Overall, the present study elucidates the extensive transcriptomic changes of ovarian cancer cells in response to LH receptor activation, which provides a comprehensive and objective assessment for determining new cancer therapies and potential serum markers, of which over 100 are suggested.

## Background

Ovarian cancer is the most lethal form of gynecological cancer. In 2009, over 21,550 new cases were diagnosed in the United States, and 14,600 of those cases resulted in death [1]. The relatively high death rate, compared to diagnosed cases, is due to the lack of an effective method for early detection. In most cases, the cancer has progressed to an advanced stage when detected, with only about a fourth of the women having the disease correctly diagnosed in a localized state. As a result, the five-year survival rate is roughly 30-40% of the diagnosed cases, independent of the therapies used [2]. Major factors, including inherited mutations in the *BRCA1 *and *BRCA2 *genes [3,4] and conditions that lead to more ovulatory periods, such as early menarche, late menopause, and nulliparity [5], have been strongly linked to increased risk of ovarian cancer development; however, the role of carcinogens and other possible contributing factors are still largely unknown [6].

It has been recognized for several years that a strong correlation exists between the risk of developing ovarian cancer and conditions such as infertility and menopause [7-9], which lead to increased exposure to the pituitary hormones, luteinizing hormone (LH) and follicle-stimulating hormone (FSH), thus targeting the gonadotropins as putative choices when investigating new therapy options, a topic that has been reviewed [6,10]. Through their regulation of granulosa, theca, and luteal cell function and differentiation, LH and FSH actions are critical for ovarian steroidogenesis, and LH is responsible for inducing ovulation [11-13]. As of now, there is only indirect evidence indicating a causal relationship of gonadotropic action and ovarian cancer development, such as a significant number of cancer cases presenting with LH receptor (LHR) expression and the increased cancer risk associated with elevated gonadotropins in serum or hypersecretion of LH [14]; the controversy still exists whether there is a direct effect of LH on ovarian surface epithelium (OSE) tumor growth, survival, and progression [2,6,10,15].

In contrast to the above considerations, there are clinical reports showing that the use of gonadotropins to treat infertility does not increase the risk of ovarian cancer, or, if so, the risk is very slight [16,17]. This controversial area, including the impact of gonadotropin ablation with GnRH analogs, was recently reviewed with the conclusion that if gonadotropins are involved in ovarian cancer, their role is probably more important in tumorigenesis and early growth, not in later stages [15]. Consistent with the clinical controversy surrounding gonadotropins and ovarian cancer, there are mixed, often conflicting, reports on established ovarian cancer cell lines regarding the actions of gonadotropins on cell proliferation, invasion, and migration [6]. Indeed, as discussed later, opposing conclusions have been reached by different groups investigating the same cell line. Consequently, a thorough examination of LH action on genetic alteration in ovarian cancer is desired in order to determine if LH contributes to any essential component of cancer development such as self-sufficiency in growth signals, evasion of apoptosis, sustained angiogenesis, tissue invasion and metastasis, etc. [18].

The goal of the present study was to ascertain if transcriptomic profiling of an ovarian cancer cell line could provide useful information on LH activation of LHR, not whether LH has any role in cancer initiation. Cultured SKOV-3 human ovarian carcinoma cells were chosen as control (LHR-)[14,19], and the experimental cells were obtained by stably transfecting the SKOV-3 cells to express about 12,000 functional LH receptors per cell (LHR+). Since we have reported elsewhere that, in *in vitro *assays [20], the LHR+ cells, but not the LHR- cells, exhibited reduced proliferation and reduced migratory and invasive properties in response to LH, the hypothesis to be tested herein is that microarray analysis can elucidate the cellular pathways that are operative in response to LH activation of LHR in these ovarian carcinoma cells, by conducting a detailed examination of the transcriptional alterations in these cells in terms of mRNA expression and functional and pathway enrichment. The results of this study have enabled us to determine the overall effects on the major pathways in the LHR+ cells and thus obtain a better understanding of LHR expression and LH-mediated LHR activation on this epithelial ovarian carcinoma cell line. In addition, over 100 proteins have been identified that warrant further studies on their potential as serum markers of LHR-positive ovarian cancer in postmenopausal women.

## Methods

### SKOV-3 Cells and Transfection

The parent SKOV-3 ovarian cancer cell line was chosen as a control in this study since it does not express LHR [14,19,20], and, following transfection, the LHR+ cells serve to determine the alterations in gene expression elicited by LH. The LHR+ cells bound [^125^I]-human chorionic gonadotropin with a K_d _of 0.3 nM (human chorionic gonadotropin and LH utilize the same G protein-coupled receptor, LHR), consistent with the binding affinity using ovarian reproductive cells, and responded to LH with increased intracellular levels of cAMP and inositol phosphates. In total, six groups of SKOV-3 cells (LHR-, LHR+, and LHR+ incubated with LH for various times: 1, 4, 8, and 20 h), each with three independent replicates, were used for examining the cell response. These times were chosen to provide temporal information on the early, intermediate, and later response genes altered by LH-mediated LHR activation.

### Microarray and PCR Experiments

Total RNA was extracted from the above 18 SKOV-3 samples [20] and was amplified using the NuGEN™ Ovation™ RNA Amplification System V2. The resultant fragmented and labeled cDNA was added to the hybridization cocktail in accordance with the NuGEN guidelines for hybridization onto Affymetrix human genome U133 Plus2 Arrays. Following hybridization for 18 h at 45°C, the array was washed and stained on the GeneChip^® ^Fluidics Station 450 using the appropriate fluidics script, before being inserted into the Affymetrix autoloader carousel and scanned using the GeneChip^® ^Scanner 3000. The microarray analyses were done by Almac Diagnostics, Durham, NC. The raw data has been deposited to GEO database (Accession ID: GSE27328).

Poly(A)+ RNA was extracted from the cells and equivalent amounts were converted to cDNA, which was then analyzed by qRT-PCR. 23 genes are tested, which are mostly associated with cell growth and invasion (Additional file 1 Table S1). The amounts of cDNA for each gene were determined in duplicate by qRT-PCR with the SYBR Green detection system, and the relative gene expression was calculated from the Ct values, where Ct is the cycle at which the threshold (i.e. the number of cycles at which the earliest measurable fluorescence signal reaches 25X baseline) can be detected in a qRT-PCR assay [21]. The relative gene expression is given as a ratio of Ct of the gene of interest to that of the housekeeping gene, GAPDH, taken as a reference gene [GAPDH-287F: GAAATCCCATCACCATCTTCC

AG; GAPDH-599R: CTTTGGTATCGTGGAAGGACTCAT] and distilled water as a negative control,

### Data Quality Control (QC) and Statistical Analyses

QC was performed for each hybridized array by assessing quality metrics comprehensively, and hierarchical clustering and principal components analysis were employed for data QC assessment by using 26,821 transcripts that passed the background filter, i.e., three times the standard deviation of the average background intensity of the 18 samples, as shown in Additional file 2 Fig. S1. The results of clustering and data reduction were assessed comprehensively to ascertain the suitability of the results for further analysis.

Subsequently, statistical analyses were performed to identify the differentially expressed genes between any two groups, especially at the transition points when LHR and LH are introduced. The ANOVA [22] and Mann-Whitney tests were initially applied, and the geometric mean of gene expression was calculated within the triple duplicates. Given the different tests underlying the individual significance, differential expression was assessed by applying p-value < 0.05 (restraining FDR < 0.1) and fold-change ≥ 2.0. More rigorously, we only focused on those expression changes consistently observed at the transition points, which means the expression levels of the triplicate measures of group A are all higher (or lower) than those of group B. Overall, the experimental design, coupled with the statistical significance and fold change criteria employed, engender high confidence of selecting reliable differential expressions. Both hierarchical clustering [23] and self organization maps (SOMs) [24] were applied to extract co-expression patterns associated with LHR expression and LH-mediated activation, especially to identify the significant functional clusters among the profiles. Enrichment analyses on functional families and pathways have been carried out according to Gene Ontology (GO) and KEGG curation [25], respectively.

### Public Microarray Data for Normal Human Ovarian Surface Epithelium (HOSE) Cells

Public normal HOSE expression dataset (GSE14001) [26] was downloaded from the GEO database for comparative purposes, which were collected using the identical Affymetrix platform. The microarray analysis was done on RNA obtained from short-term cultures of three different normal human ovarian surface epithelial cells that were initiated from the surface scraping of normal ovaries [26]. The same RMA algorithm was applied for gene expression summarization; no further normalization was conducted between different cell types to retain the variance of overall mRNA expression.

## Results

In earlier *in vitro *studies [20], it was shown that, when compared to LHR- cells, LHR expression, in the absence of added LH, had no effect on cell proliferation, although it did reduce the invasiveness when measured using Matrigel to mimic the basement membrane; moreover, the degree of wound closure, a measure of migration using a scratch assay, was increased by 0.5% fetal bovine serum in the LHR+ cells. The addition of LH to the LHR+ cells, but not the LHR- cells, reduced the growth rate and migratory properties, but there was no further reduction in the invasive index compared to that elicited by LHR alone. Herein, we examined the corresponding gene expression changes, with one of the goals to identify mRNA expression patterns that are correlated with the altered cell characteristics.

### Altered Gene Expression and Coexpression Patterns

**Figure 1:**
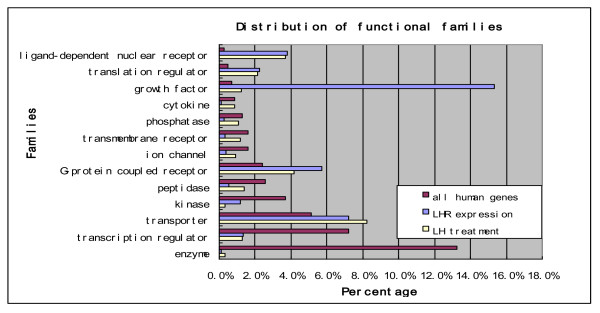
**Distributions of the 2,373 differentially expressed genes in SKOV-3 cells across IPA functional families**. Each blue bar represents the percentage of differentially expressed genes associated with LHR expression; each yellow bar represents the percentage of differentially expressed genes upon incubation with LH; each red bar is the percentage of all human genes. The x-axis represents the percentage and the y-axis denotes functional families.

A total of 54,671 transcripts were originally profiled, among which 2,373 genes exhibited at least 2-fold differential expression between any two experimental groups (see detailed statistics in Additional file 1 Table S2), including 1,783 genes differentially expressed in LH-treated cells. Out of the 23 differentially expressed genes analyzed by qRT-PCR in this study and earlier [20], we found that 22 genes exhibit consistent expression pattern between microarray and qRT-PCR data (Additional file 1 Table S1), which indicate that majority differential information derived from microarray is reliable. According to the IPA annotation [27], 689 differential genes are cancer-related, and 265 genes are highly expressed in the ovary (see Additional file 1 Table S3). Five major functional families were found to be significantly enriched by the differentially expressed genes, including growth factors, translation regulators, transporters, G-protein coupled receptors, and ligand-dependent nuclear receptors (Figure 1). Generally, these differentially expressed genes participate in pathways involved in the cell cycle, focal adhesion, cytokine-cytokine receptor interaction, regulation of the actin cytoskeleton, purine metabolism, and a number of key signaling pathways such as MAPK, TGF-β, p53, and Jak-STAT.

**Figure 2:**
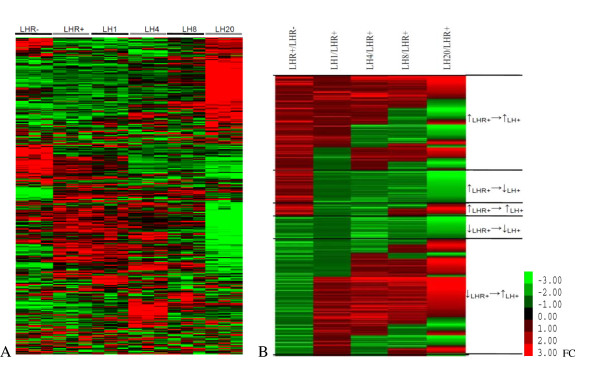
**A. A heatmap showing normalized gene-expression profiles for SKOV-3 cells under six conditions**. B. A heatmap showing gene-expression patterns under different regulation (for each group, the mean expression value of the three replicates is shown, and the five transitions are LHR+/LHR-, LH1/LHR+, LH4/LHR+, LH8/LHR+ and LH20/LHR+), where LHR- denotes the control or mock-transfected SKOV-3 cells, and all others are the LHR+ cells with no LH added or after incubation with LH for 1, 4, 8, and 20 h, respectively.

The 2,373 genes were subject to hierarchical clustering [23] for identification of distinct gene-expression patterns across all sample groups. In Figure 2A, different expression patterns were observed across the five different transitions, i.e. LHR-/LHR+ and LHR+/LHR+ plus LH for each of the four time points, clearly supporting the hypothesis that LHR expression and LH-mediated receptor activation impose significant effects on gene expression in ovarian cancer cells.

In total, 12 highly correlated expression patterns were identified from the differentially expressed genes, by using a self organization map (SOM) [24] (Table 1). The gene lists of each cluster are given in Additional file 1 Table S4. Seven clusters (C1-C7) represent the up-regulated genes, while the other five show down-regulated genes concomitant with LHR expression in the cells. After incubation of LH with the LHR+ cells, the genes of each group became more diverse in terms of their expression level changes, either being up-regulated, down-regulated, or unchanged, falling into five categories. Within each cluster, enriched GO and pathways were identified (Table 1), reflecting the major involved functional groups or cellular processes, as discussed in the next two sections.

**Table 1:** 12 gene clusters identified from the differentially expressed genes

Categories	Clusters LHR-,LHR+,1h,4h,8h, 20h	#. of genes	GO(s) enriched	Pathways enriched
	1	144	extracellular matrix structural constituent platelet-derived growth factor alpha-receptor activity regulation of vesicle fusion hydroxyacid-oxoacid transhydrogenase activity	TCR EGFR1
	
LH(↑)/LHR(↑)	2	157	negative regulation of apoptosis leukocyte differentiation carboxylic acid metabolic process	EGFR1 TGFBR ID KitReceptor
	
	3	152	multicellular organismal development cell proliferation cyclic-nucleotide phosphodiesterase activity regulation of transcription, DNA-dependent cell-cell signaling	Hematopoietic cell lineage

LH(-)/LHR(↑)	4	205	nervous system development neurogenesis notch binding calcium ion binding cell morphogenesis	NOTCH

	5	157	response to external stimulus positive regulation of cellular metabolic process	AndrogenReceptor EGFR1
	
LH(↓)/LHR(↑)	6	270	cadmium ion binding transcription spermidine biosynthetic process regulation of RNA metabolic process	MT-HeavyMetal-Pathway TCR IL4 TNF alpha/NF-kB
	
	7	167	neutrophil chemotaxis positive regulation of heart rate calcium-mediated signaling leukocyte chemotaxis regulation of cell migration	IL-7 ID

LH(↑)/LHR(↓)	8	145	extracellular region collagen fibril organization complement component C3b binding fibrillar collagen inflammatory response response to external stimulus protein digestion	IL-7 Wnt
	
	9	261	amylase activity calcium ion binding homophilic cell adhesion synaptogenesis	IL-7 EGFR1

LH(-)/LHR(↓)	10	288	proteinaceous extracellular matrix polysaccharide binding glycosaminoglycan binding regulation of defense response G-protein signaling, coupled to IP3 second messenger enzyme inhibitor activity	Wnt EGFR1

LH(↓)/LHR(↓)	11	71	regulation of aldosterone metabolic process regulation of hormone metabolic process auditory receptor cell differentiation epidermis development growth factor activity	NOTCH TGFBR
	
	12	191	cell cycle phase mitosis microtubule cytoskeleton	BCR

Plots in the 2nd column represent the expression pattern of six conditions (SKOV-3 control, i.e. mock-transfected (LHR-), LHR expression but with no added LH, and incubation of the LHR+ cells with LH for 1h, 4h, 8h, and 20h) "↑" and "↓" denote responses of up-regulation and down-regulation, respectively and "-" denotes no alteration of gene expression.

### Genes regulated by the Presence of LHR and Relevant Pathways

Of the 414 genes that were differentially expressed significantly when LHR was introduced, 144 were up-regulated and 270 down-regulated. A few pathways, including gap junction, purine metabolism, calcium signaling, and actin cytoskeleton regulation, are associated with the up-regulated genes, perhaps indicating a moderate activation of these processes. Since one of the objectives is to examine the regulation of the genes that may promote or inhibit tumor growth, migration, and invasiveness in LHR+ tumors, the up-regulated *TUBAL3, TUBB2B*, and *GUCY1B3 *genes involved in gap junction formation and function may indicate a reduced tumor progression and metastasis [28]. Opposing these increased expressions, LHR+ cells exhibit significant down-regulation of genes associated with cellular processes such as cell communication, ECM-receptor interaction, regulation of vesicle fusion, and focal adhesion, for example genes encoding extracellular matrix structural constituents (*KRT7, DSC3, KRT16, TNC, LAMB3*), collagens (*COL3A1, COL6A3, COL4A1, COL1A2*), and matrix metalloproteinases (*MMP1, MMP2, MMP13, MMP14*). The negative effect on cell communication and ECM interaction is consistent with a reduced invasive activity of the cancer cells, thus inhibiting cancer progression. In addition, other down-regulated genes are found relevant to apoptosis (*PIK3R3, IL1R1, FAS, TNFSF10*) and major signaling pathways (*P53, TGF-β, ERBB HER-2*).

### Responses of Gene Expression and Pathways Following LH-Mediated LHR Activation

A total of 1,783 genes were found to be regulated by LH, when compared to gene expression in LHR+ cells (including all clusters in Table 1 except for C4 and C10). The up-regulated genes are enriched in 21 pathways (Table 2), including VEGF signaling, gap junction, and immune responses (the Toll-like receptor signaling and the B cell receptor signaling pathways). Besides the immune responses that are generally observed in most types of cancers, the activation of genes involved in VEGF signaling may be hypoxia responsive and introduce a positive effect on cancer growth, while those involved in gap junction and Notch signaling accelerate cell-cell communication and influence several key aspects of the normal development by regulating differentiation, proliferation, and apoptosis [29].

**Table 2:** Pathways significantly enriched by differentially expressed genes regulated by LH (p-value < 0.5)

21 pathways uniquely involved in the up-regulated genes					
**Pathway (up-regulated genes involved)**	**LHR+**	**LH1**	**LH4**	**LH8**	**LH20**

Gap junction	3				5

Melanogenesis		3	5	4	5

Toll-like receptor signaling pathway		3			8

Epithelial cell signaling in Helicobacter pylori infection		3	4		3

VEGF signaling pathway			4		4

Adherens junction			5	3	3

B cell receptor signaling pathway			3		6

Adipocytokine signaling pathway			3		4

Hedgehog signaling pathway			3		

Basal cell carcinoma			3		

Cell adhesion molecules (CAMs)					3

Long-term potentiation					3

Glutathione metabolism					5

Long-term depression					4

Androgen and estrogen metabolism					4

Dorso-ventral axis formation					3

mTOR signaling pathway					3

Metabolism of xenobiotics by cytochrome P450					5

Arachidonic acid metabolism					3

Fc epsilon RI signaling pathway					6

GnRH signaling pathway					3

22 pathways uniquely involved in the down-regulated genes					

**Pathway (down-regulated genes involved)**	**LHR+**	**LH1**	**LH4**	**LH8**	**LH20**

Cell cycle	**3**		**11**	**3**	**14**

p53 signaling pathway	**3**				**10**

Complement and coagulation cascades	**7**				**4**

Pyrimidine metabolism					**8**

Alanine and aspartate metabolism					**5**

Urea cycle and metabolism of amino groups					**6**

Valine, leucine and isoleucine degradation					**4**

Propanoate metabolism					**3**

Neurodegenerative Diseases					**3**

Pyruvate metabolism					**3**

Alkaloid biosynthesis II					**3**

Glycerolipid metabolism					**3**

Carbon fixation					**3**

SNARE interactions in vesicular transport					**3**

beta-Alanine metabolism					**4**

Arginine and proline metabolism					**4**

Methionine metabolism					**3**

Selenoamino acid metabolism					**3**

Aminoacyl-tRNA biosynthesis					**4**

Phenylalanine metabolism					**5**

Glutamate metabolism					**3**

Basal transcription factors					**3**

34 pathways involved in both up- and down-regulated genes					

**Pathway (both up-/down-regulated genes involved)**	**LHR+**	**LH1**	**LH4**	**LH8**	**LH20**

MAPK signaling pathway	4	8	9	6	15

	**3**		**3**		**12**

Apoptosis					7

	**4**				**6**

Focal adhesion		3	6	4	21

	**12**				**9**

Regulation of actin cytoskeleton	3	3	5		16

	**3**		**3**		**7**

TGF-beta signaling pathway		4	7	4	7

	**3**				**4**

Cell Communication					11

	**13**				

ECM-receptor interaction					15

	**10**				

Jak-STAT signaling pathway		4	6		8

				**3**	**4**

Tight junction			3		4

					**4**

ErbB signaling pathway			3		5

	**4**				**4**

Wnt signaling pathway			4		

					**7**

PPAR signaling pathway					3

					**3**

Purine metabolism	3		3	3	4

					**8**

Cytokine-cytokine receptor interaction		9	13	5	12

	**8**		**3**		**8**

Axon guidance			6	3	11

	**4**				**6**

Prostate cancer		3	5	3	8

					**5**

Hematopoietic cell lineage			5	6	7

	**3**				

Natural killer cell mediated cytotoxicity			4		6

	**4**				

Neuroactive ligand-receptor interaction			3		3

	**6**				**3**

Calcium signaling pathway	3			3	6

	**4**				**7**

Insulin signaling pathway			4		5

					**6**

Glycerophospholipid metabolism			4		5

					**3**

Complement and coagulation cascades			3		7

	**7**				**4**

Tryptophan metabolism			3		5

					**4**

T cell receptor signaling pathway			3		6

					**3**

Starch and sucrose metabolism					3

	**4**				**3**

Leukocyte transendothelial migration					11

	**5**				**4**

Nitrogen metabolism					6

					**3**

Phosphatidylinositol signaling system					3

					**3**

Tyrosine metabolism					3

					**6**

Histidine metabolism					3

					**4**

Ubiquitin mediated proteolysis			3		

					**9**

Glycan structures - biosynthesis 1					3

	**3**				**4**

Glycine, serine and threonine metabolism					3

					***6***

The number in each cell represents the number of differential genes involved in the corresponding pathway. (Note: the top areas designate up-regulated genes, while the underlined (bold) one (bottom) signify down-regulated genes.)

The genes that are down-regulated by LH represent 22 pathways (Table 2). Besides the continuous inhibition of the cell cycle, p53 signaling, and the complement and coagulation cascades, LH also seems to impose a negative and delayed effect on a few metabolic pathways related to pyrimidine, glycerolipid, methionine, androgen, and estrogen metabolism. These results indicate an LH-mediated reduction in certain aspects of nucleic acid, lipid, and amino acid metabolism. Since the epithelial cells are not steroidogenic, the down-regulation of androgen and estrogen pathways may relate more to sex steroid action [30,31].

**Figure 3:**
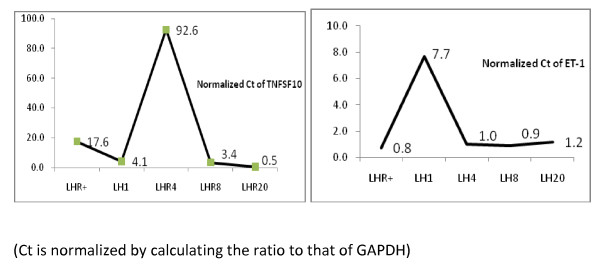
**qRT-PCR measures of gene TNFSF10 and ET-1**. The left panel shows the normalized Ct of TNFSF10 (primer sequence (F) AAGACTGTCAGCTTCCAAACATTAA(R) GTGATACACTACT TGAGAGATGGAT) and the right panel shows the normalized Ct of ET-1 (primer sequence (F) AGGCCCTGAGTTGGCAGTGGCCCAT (R) ATGGGCCACTGCCAACTCAGGGCCT).

Table 2 shows 34 additional pathways consisting of both up- and down-regulated genes to different extents, among which the overall effects on tumor growth and apoptosis cannot be evidently inferred. For a few, one particularly interesting observation is the substantially increased expression of the tumor necrosis factor member 10 gene (*TNFSF10*), involved in natural killer cell-mediated cytotoxicity (Figure 3). *TNFSF10 *encodes the cytokine tumor necrosis factor-related apoptosis-inducing ligand (TRAIL) that binds to TNF and induces apoptosis, primarily in tumor cells [32].

### Genes Most Highly-Expressed and Most Differentially-Expressed

**Figure 4:**
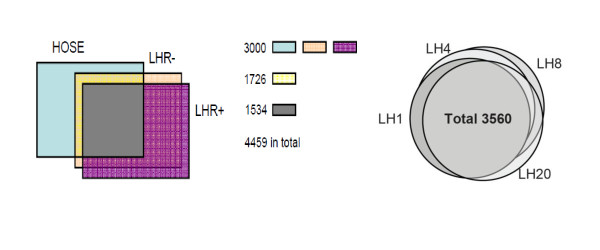
**Venn diagram of the top 3,000 differentially expressed genes from HOSE cells, mock-transfected SKOV-3 cells (LHR-), and LHR+ cells (LHR) (left), and temporal effects after addition of LH to the LHR+ cells (right), respectively**.

To exclude the possibility that some effects of LH on cell growth and apoptosis were masked by the extremely high levels of gene expression in SKOV-3 cells, the most highly-expressed genes (top 5%, ~3000 transcripts) in each group were examined and compared with those from normal HOSE cells. Figure 4 shows that the genes most highly expressed in SKOV-3 cells and normal HOSE cells are largely different, with only 1,726 out of the 3,000 transcripts in common. The 1,056 unique genes, specific in the LHR-SKOV-3 cells, are participating in the regulation of translation, cell division, chromosome partitioning, post-translational modifications, protein turnover, chaperones, and signal transduction mechanisms, indicating possible alterations of these processes in cancer compared to normal HOSE cells, such as an increase in the overall rate of protein synthesis and translational activation of the mRNA molecules involved in cell growth and proliferation. The majority (2,748) of the highly expressed genes in the LHR- SKOV-3 cells continue to be expressed in the LHR+ cells.

In contrast, the highly-expressed genes are quite consistent in the LHR+ cells incubated with LH, where 3,560 genes were involved. The 689 genes specifically introduced by LH-mediated LHR activation, compared to LHR+ cells, reveal the involved cellular processes such as coenzyme metabolism, post-translational modifications, nucleotide transport, DNA replication and repair, intracellular trafficking, and secretion.

In addition, the most differentially expressed genes were examined (Additional file 1 Table S5), and a few were found to be altered significantly by LHR expression, such as *ERBB4 *(↓46) and *CASP1 *(↓44). Down-regulation of *ERBB4 *is deemed to be beneficial as its over-expression may promote cell proliferation, while down-regulation of *CASP1 *may result in a suppressive effect on cell apoptosis [33]. Following LH activation of LHR, the most highly up-regulated genes, e.g., *PDE4B, TNFSF10, FOSB*, and the highly induced early and intermediate response genes, e.g., *THBS1, CCl20, DUSP1*, are found to occupy a gene network connecting transcriptional regulation, cell proliferation and differentiation, apoptosis, and multiple signaling transductions such as MAPK, Erk1/Erk2 MAPK, Jak-STAT, VEGF, and the TGF-β signaling pathway. Thus, from some of the results one could argue that LH may serve as a positive regulator on cancer growth and invasion through overexpression of *CCl2 and FOSB*. However, the large increase in the expression of *TNFSF10 *can act to increase apoptosis. The high level of up-regulation of *PDE4B *is interesting since the enzyme, a cyclic nucleotide phosphodiesterase up-regulated by cAMP [34], is responsible for inactivating cAMP and thus rendering the cells refractory to additional LH signaling for an extended time.

### Major Pathways Altered in the LHR+ and LH+ SKOV3 Cells

Ovarian carcinogenesis is a complicated process that involves the deregulation of multiple signaling pathways. In this study, proteins and signaling pathways involving Wnt signaling, p53 tumor suppressor, APC/β-catenin signaling, K-Ras concogene, and EGFR tyrosine kinase were found to be affected by LH activation of LHR, either positively or negatively. Of particular interest was the result demonstrating that LH dramatically activates the expression of the interleukin-6 gene (*IL-6*) (33↑), a pleiotropic cytokine that is assumed to be involved in ovarian carcinogenesis and may induce signaling pathways such as toll-like receptor, NOD-like receptor, cytosolic DNA-sensing, and Jak-STAT [35,36], which, in this sense, may indicate a potential therapeutic target for treating ovarian cancer. Moreover, an increase in the production of potent growth factors like IL-8 may facilitate tumor growth and angiogenesis [37].

To sum up the major impact of LH, we have grouped all involved pathways (Additional file 1 Tables S6-7) according to their cellular functions (Table 3). A few of them were selected for a closer examination (cf. Discussion), which include the cell cycle, the MAPK pathway, apoptosis, the Jun and Fos family of transcription factors, and other signaling pathways.

**Table 3:** Number of altered pathways contributing to a general cell function (The detailed of each pathway can be found Additional file 1, Table S6-7)

General function	Number of pathways involved
Signaling	34

Receptor interaction	3

Metabolism	31

Junctions	3

Disease	23

Immune system	19

Apoptosis	14

Cell cycle	9

Gene expression/regulation	11

Miscellaneous	41

### Identification of Potential Molecular Markers

**Figure 5:**
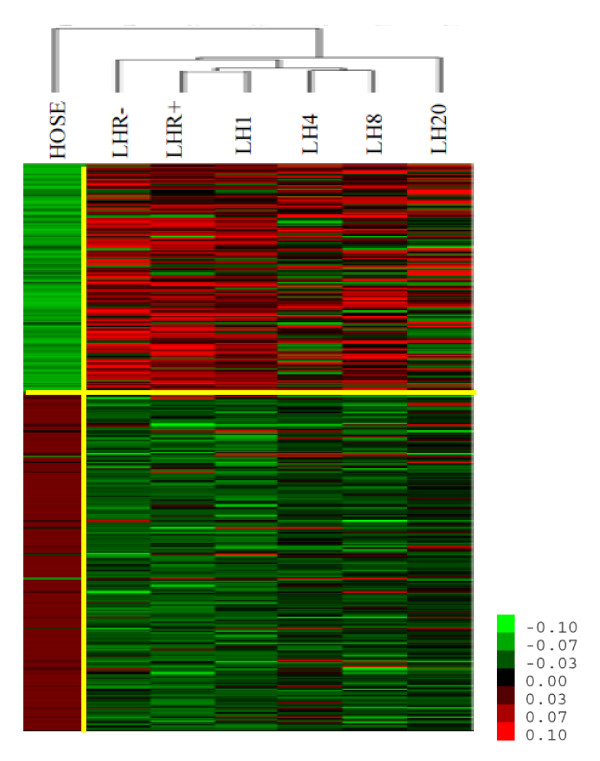
**A heatmap of the 185 and 248 genes, up-regulated and down-regulated, respectively, in normal HOSE versus SKOV-3 cells: control (LHR-) and LHR+ receiving no LH (LHR+) and following incubation with LH for 1, 4, 8, and 20 h**.

A comparison of the gene expression profiles between normal OSE cell and SKOV-3 cells (including all LHR+ cancer cells) has the potential to identify a group of genes that can discriminate between normal and cancer cells regardless of LHR expression and LH action. Two lists of genes have been identified as up-regulated (185 genes) and down-regulated (248 genes) in all cancer versus normal cells, whose expression profiles are shown in Figure 5 (see names in Additional file 1 Table S9). Functional analysis reveals that the up-regulated genes are involved in cell communication, ECM-receptor interaction, and focal adhesion, especially functioning in cell division and chromosome partitioning, as well as carbohydrate transport and metabolism, which are fundamental processes for cancer growth. We have conducted the specificity analyses of the identified markers against public microarray gene-expression data for other human diseases (http://bioinfosrv1.bmb.uga.edu/DMarker/) and obtained 106 genes whose differential expression are specific to ovarian cancer. Among these genes, nine have been reported with the same expression changes in a newly-developed YDOV-157 cell line versus HOSE (Additional file 1 Table S9), which illustrate some consistency between different cell lines. These results engender confidence in proposing some genes as potential molecular markers to discriminate between ovarian epithelial carcinoma cells and normal OSE cells. Based on a recently developed approach from this laboratory [38], 103 of these genes (Additional file 1 Table S9) were predicted in which their protein products may be secreted into the bloodstream, thus providing another important pool of potential serum markers for further investigation. According to the proteomic reports from the Plasma Proteome Project (PPP) [39] and a literature search for diseased protein markers [38], we know that 22 of these proteins have been identified as secreted proteins in normal or diseased blood (Additional file 1, Table S9). While it is unlikely that just one marker would emerge with good specificity and sensitivity, combinations of two or more may prove highly useful. Some of the predicted proteins could be peptides/fragments derived from extracellular matrix proteins and membrane receptors, many are readily soluble and assayable, e.g. chemokine ligands 1, 5, 9, 10, 11, and 18, placental growth factor (PGF), and growth hormone secretagogue receptor ligand (GHRL), to mention but a few.

### LH Regulation on Known Therapeutic Targets

Our literature search against the Therapeutic Target Database (TTD) [14] found that 48 therapeutic targets were reported to be ovarian cancer-associated, including 18, 20, and 12 targets in three categories, successful, clinical trials and, research, respectively (detailed list in Additional file 1, Table S10). Our data cover 39 of the 48 therapeutic targets, some of which are significantly regulated by LHR activation. Table 4 lists four of these targets with the greatest changes in gene expression.

**Table 4:** Illustration of the reported therapeutic targets that are regulated by LHR activation

Type	Target name	LHR+	LH1	LH4	LH8	LH20
Research	Endothelin-1 (ET-1)	-1.1	10.2	4.3	1.8	2.1

Research	Stromal cell-derived factor 1 (SCD-1)	1.2	1.6	10.3	6.3	4.2

Research	Insulin-like growth factor II (IGF2)	-8.7	8.6	11.6	3.9	9.2

Clinical trail	Kinesin-like protein KIF11 (KIF11)	-1.5	-1.1	-2.3	-1.3	-1.6

Fold changes are shown; the category (successful, clinical trial or research) indicates the different phase of the therapeutic targets discovery.

Endothelin-1 (ET-1)-mediated activation of the endothelin-1 receptor is known to result in vasoconstriction and smooth muscle cell proliferation and is implicated in the pathogenesis of hypertension, coronary vasospasms, and heart failure. More recently, it has been shown that ET-1, acting through its G protein-coupled receptor, ET_A_R, is an important component of ovarian cancer initiation and progression [40-42]. These findings have led to an interest in the development of endothelin-converting enzyme-1 inhibitors and small interfering RNAs as a new therapeutic agent for ovarian cancer [43]. Interestingly, the LHR+ cells respond to LH with a 10-fold increase in ET-1 gene expression, peaking at 1 h and remaining slightly elevated up to 20 h. The LH-mediated increase in ET-1 gene expression was confirmed by qRT-PCR (Figure 3). ET_A_R expression is also increased about 2-fold in response to LH, while there are no significant effects on expression of the genes for endothelin-converting enzyme-1 and the endothelin B receptor. These results alone could indicate a possible enhancement of cell proliferation in response to LH. LH-mediated LHR activation also significantly up-regulates the stromal cell-derived factor 1 (*SCD-1*) and insulin-like growth factor II (*IGF2*) genes. The former has been reported to increase the invasiveness and migration of breast cancer cells [44], and the latter is known as a fetal promoter of cell proliferation that is involved in various forms of cancer [45]. The up-regulation of just these genes could suggest that LH exerts positive effects on tumor growth and metastasis. We know, however, from the experimental evidence that the up-regulation associated with these growth-promoting genes is not manifested in LH-activated LHR+ cells, and thus expression of the other negative regulators, e.g. *c-JUN, TNFSF10, and MMPs*, must assume a dominant role in relating gene expression and tumor cell properties.

## Discussion

This work presents data obtained using a novel epithelial carcinoma cell model for studying the response to LHR expression and activation in ovarian cancer, mimicking a significant percentage of tumors that arise in postmenopausal women, i.e. characterized by LHR expression and high circulating concentrations of LH. Overall, the results showed dramatic changes in the transcriptome elicited by the expression of LHR in SKOV-3 cells with no added ligand and following addition of LH to the LHR+ human ovarian cancer cells. The expression of LHR, in the absence of LH, altered the transcription of 414 genes. This result could arise from a small increase in signaling, e.g. via protein kinases A and C, if the receptor infrequently adopts an active conformation; there may also be some degree of ligand-free signaling of LHR. Functional and pathway analyses revealed both positive and negative effects of LH-mediated LHR activation on LHR+ SKOV-3 cell growth and apoptotic pathways. Since we know from earlier studies that LH addition to the LHR+ SKOV-3 cells led to an inhibition of growth over a 7-day time course and that LH acted to reduce invasion and migration in short-term *in vitro *assays [20], one can speculate that the dominant transcriptomic changes leading to the observed cellular phenotype in response to LH could involve up-regulation of *TNFSF10, TUBAL3, and TUBB2B*, as examples, and down-regulation of *c-JUN *and the *MMPs*, again as examples.

Conflicting reports on various ovarian carcinoma cell lines have appeared, perhaps reflecting to some extent the heterogeneous nature of ovarian cancer as reflected in the cell lines used [6], as well as possible further dedifferentiation of the cells in long-term culture. For example, there are several reports on SKOV-3 cells showing the presence of LHR by Western blots and responses to LH, including increased: cell proliferation (at 0.1 and 1 μg/mL, but not at 10 ng/mL) and invasiveness, MMPs 2 and 9, cyclooxygenases 1 and 2, and AKT, and decreased tissue inhibitor of metalloproteinase-1 [46,47]. However, other reports, based on PCR, [^125^I-hCG] binding, and immunohistochemistry, failed to detect LHR in SKOV-3 cells [14,15,20]; moreover, 0.1 μg/mL of hCG [19] and 0.5 μg/mL of LH [20] did not lead to increased proliferation. These discrepancies are not easily explained, other than the real possibility that different variants exist in SKOV-3 cells, arising perhaps from long-term culture and passage number. Hence, each report in which the presence or absence of LHR is documented must be based on its own merit. For the studies reported herein, we have confidence that the mock-transfected SKOV-3 cells used do not express LHR, while the transfected cells express a functional gonadotropin receptor.

To examine if the LH-mediated alteration in gene expression is specific to SKOV-3 cells, we compared the gene expression changes reported in other cell types include human cumulus cells [48], granulosa lutein cells [49], and granulosa cells [50], all modulated by LH or FSH (Additional file 1, Table S11). Among our so-identified differentially expressed genes that also overlap with those reported in each of the above studies, only a small portion of genes, say 7 (out of 21), 4 (out of 6), and 5 (out of 23), shows consistent alteration in SKOV-3 versus other cells. Another study reported human LH (2-4 μg/mL) and CG (10 IU/mL) consistently evoked oscillatory calcium signals in HEK293 cells transfected with the human LH receptor [51]. Our findings that the moderately activated calcium-mediated signaling pathway and the calcium ion-binding pathway reflected by the up-regulated genes may suggest a similar effect in SKOV-3 cells, which needs further investigation. These studies suggest that LH-mediated LHR activation impacts on various types of cells, but some of the alterations identified in the current study are more specific to SKOV-3 cancer cells.

An analysis based on transcriptomic profiling alone is far from adequate to accurately conclude the overall effects of LH-mediated LHR activation on ovarian cancer, but this work exemplifies the gargantuan amount of information and cellular responses associated with LHR expression and activation in ovarian epithelial cancer cells. The involvement of several pathways deserves further elaboration.

***Gap junction ***gene alterations were revealed by a number of up-regulated connexins that are known to function as tumor suppressors, regulating cell growth, differentiation, and, possibly, metastasis. The most highly expressed genes of the connexins, including *GJA1, GJA3*, and *GJA7*, were moderately up-regulated. It is known that cancer cells frequently exhibit down-regulation of gap junction proteins [52], and chemopreventative treatments to increase connexins offer improved anticancer activity; thus, viewed from the up-regulation of connexin genes, LH may exhibit a moderate negative effect on cancer growth and migratory properties. This observation is consistent with the finding that LH reduces the growth rate, migration, and invasiveness of LHR+ SKOV-3 cells [20].

***Apoptosis ***plays an important role in cancer development and is closely associated with the cell cycle. In the present research, 61 of the 2,373 genes of interest were found to participate in apoptosis, and their functions range from promoting or inhibiting the pathway. There are minimal effects on the most apoptosis-related genes, including *P53, P21, BCL-2, BAX*, and *BAD*, but significant down-regulation of some other genes, such as *CACSP1, PPP1R15A, PLEKHF1, BMF TRAF3*, and *FAS*, may indicate a moderate inhibition of apoptosis. This possibility, of course, needs further investigation. These results complement and extend the observations of others on LHR+ OVCAR-3 cells, in which it was shown that LH inhibited cisplatin-induced apoptosis by increasing the expression of the IGF gene but not those for BCL-2 and BAX [53].

***Natural killer cell-mediated cytotoxicity ***was selected because of its involvement in cancer treatment and its inclusion of the six differentially expressed genes such as *TNFSF10*, one of the most highly up-regulated genes observed in this study. *TNFSF10 *has been studied extensively in relation to human cancer because of its cytotoxic effects on tumor cells. Its encoded protein, TRAIL, can bind to members of the TNF superfamily and induce apoptosis. The dramatic up-regulation of *TNFSF10 *in the presence of LH may indicate enhanced apoptosis. However, many transformed cell lines have also shown resistance to the effects of the protein, despite expressing the appropriate receptors [54]. Tumor cell survival may be due, in part, to the inhibition of TRAIL activity, e.g. to the expression of osteoprotegerin (OPG) [55]. More studies need to be performed to infer the effectiveness of this up-regulation on apoptosis of ovarian tumor cells. Another oncogene, *VAV3*, known to regulate cell growth and androgen receptor activity in prostate cancer [56], also showed a significant increase with LH addition. These two alterations strongly suggest that further studies on the LH-induced effects of natural killer cell-mediated cytotoxicity are warranted on ovarian cancer.

***The Jun and Fos family of transcription factors ***are discussed because of the observed effects that LHR expression and LH-mediated activation had on their gene expression, as well as their integral roles in cancer development. The oncogene, *c-JUN*, was found to play a role in promoting the cell cycle through stimulation of Ras, specifically activating crucial cell-cycle regulators and thus inducing the G1-S transition and enhancing cancer development and progression. Other members of the *Jun *family, such as *JUNB *and *JUND*, were found to have opposing functions to that of *c-JUN*, and in most cancers are observed to exhibit decreased expression [57]. In the present study, *c-JUN *expression was decreased after 20 h of incubation with LH, while the expression of *JUNB *and *JUND *showed only marginal changes. The importance of *JUN *expression in cancer development may indicate that its down-regulation could provide beneficial effects in controlling cancer and that the consequential up-regulation of a few less important cancer-promoting genes may be tolerated in view of the overall benefits achieved by controlling *JUN*. However, highly significant changes in *FOS *(16↑) and *FOSB *(61↑) after 1 h of LH treatment indicate an enhanced regulation in cell proliferation, malignant transformation, and invasion. It has been reported that relatively high concentrations of LH increases proliferation and invasiveness of SKOV-3 cells [46,47], which is somewhat surprising in view of the absence of measureable LHR expression in these cells [14,19,20]. An opposite finding was observed in LHR+ SKOV-3 cells [20]. Again, further investigations are needed to elucidate the overall effects of LH regulation on ovarian cancer.

***The cell cycle ***was chosen because of its importance in the regulation of cell proliferation, whose control mechanisms are often altered in cancer, leading to aberrant cell growth [58]. In the present study, most of the cell cycle-related genes were found to be involved at the G1/S and G2/M transitions and were down-regulated by LHR expression and activation. Other stages of the cell cycle in which altered gene expression was found include chromosome segregation, anaphase, mitotic spindle localization, and the spindle checkpoint. The down-regulation of cell cycle-associated genes is consistent with a reduced proliferation rate in the presence of LH.

***MAPK ***pathways are involved in the regulation of several physiological responses, such as cell proliferation, apoptosis, cell differentiation, and tissue development. Earlier studies have implicated the involvement of the MAPK cascade in carcinogenesis after linking the constitutive activity of MAPK proteins to be associated with cell transformation [6]. Therefore, the MAPK pathway has been considered a target pathway for cancer therapy [59]. Our data analysis revealed that, for each transition, the MAPK cascade had significant changes in gene expressions, as shown in Additional file 1, Table S8. However, it is inconclusive whether the LH effect through the MAPK pathway is growth enhancing or suppressing based on the gene expression data alone. The results obtained herein and in the earlier study [20] on the LHR+ SKOV-3 cells are consistent with reports that hCG is protective for breast cancer [60]. Others, working with breast cancer MCF-7 cells, provided evidence that hCG decreases the proliferation and invasiveness of these cells by inhibiting NF-κB and AP-1 activation [61]. Clearly, further studies are needed on a variety of ovarian carcinoma cell lines and primary cells to sort out the role of LH and hCG on cellular properties. From the data available, it seems unlikely that LH is tumorigenic for ovarian cancer [15], but its actions in cellular proliferation, invasiveness, and migration remain controversial.

Ongoing and additional studies are required to enable transcriptomic profiling to be useful as a diagnostic technique and as a template for treatment and biomarker discovery. The results presented herein represent but one example of many showing that microarray results alone, while providing extremely valuable information, often gives conflicting suggestions of cellular properties and always require functional studies to sort out the dominant pathways. One of the major findings of this work was the observation that > 100 proteins may be secreted into circulation in response to LH activation of the LHR in the SKOV-3 cells. This prediction, based on our documented approach [38,62], warrants further investigation in a diligent effort using clinical samples and these suggested proteins in an attempt to discover new biomarkers for ovarian cancer.

## Conclusions

Using LHR+ SKOV-3 ovarian cancer cells, our studies have demonstrated that the presence of LHR and its activation by LH results in differential expression of over 2,370 genes, leading to alterations in myriad cellular pathways. Up-regulation of several genes is consistent with the measured cellular responses to LH, i.e. a reduction in proliferation and migration. Further, over 100 proteins are suggested as potential serum markers in LHR+ ovarian neoplasms in the presence of LH, e.g. a large majority of post-menopausal women.

## Competing interests

The authors declare that they have no competing interests.

## Authors' contributions

JC performed the analysis and drafted the manuscript. BMM and JBE contributed for the pathway analysis and discussion. SWW performed cell culture experiments and PCR, and PD helped with the PCR validation. YX participated in the discussion and revision the manuscript. DP conceived the design, provided financial support, and revised the manuscript. All authors read and approved the final manuscript.

## Pre-publication history

The pre-publication history for this paper can be accessed here:

http://www.biomedcentral.com/1471-2407/11/280/prepub

## Supplementary Material

Additional file 1 **Supplementary tables**. Table S1 23 differentially expressed genes that are used for PCR validation. Table S2 Statistics of genes with their geometric mean fold-change > = 2 across any time course. Table S3 Identified 2373 differentially expressed genes, among which 689 are reported to be cancer related. Table S4 12 expression patterns that identified in 2373 differentially expression genes, which is corresponding to the blue plots in Table 1 in the main text. Table S5 The eight most significantly down-regulated genes with introduction of the LH receptor, and affected pathways. Table S6 Pathways impacted by LHR introduction (fold-change of gene expressed indicated in parentheses). Table S7 Pathways impacted by LH treatment (fold-change of gene expressed indicated in parentheses). Table S8 Expression differentiation of the genes involved in the MAPK pathway. Table S9 Two lists of genes including 185 and 248, respectively, which are identified as highly-expressed and under-expressed in all cancer cells versus normal cells. (The 106 genes that are specific to ovarian cancer and 103 that are predicted to be blood secreted are indicated). Table S10 48 therapeutic targets reported to be ovarian cancer-associated (expression fold-changes are shown; "-" indicates that the corresponding genes are not included in the microarray chip platform). Table S11 Differential gene expression compared to those observed in other cells modulated by LH or FSH. Click here for file

Additional file 2 **Supplementary figures**. Figure S1 Evaluation of the Microarray data quality and. Figure S2 qRT-PCR measures of gene TNFSF10 and ET-1 Click here for file
