# Supplementary material for: Regulation of gene expression in ovarian cancer cells by luteinizing hormone receptor expression and activation
Source: BMC Cancer. 2011 Jun 28;11:280. doi: 10.1186/1471-2407-11-280 (PMC3141782; doi:10.1186/1471-2407-11-280)
Supplement: Additional file 1 — Supplementary tables. Table S1 23 differentially expressed genes that are used for PCR validation. Table S2 Statistics of genes with their geometric mean fold-change > = 2 across any time course. Table S3 Identified 2373 differentially expressed genes, among which 689 are reported to be cancer related. Table S4 12 expression patterns that identified in 2373 differentially expression genes, which is corresponding to the blue plots in Table 1 in the main text. Table S5 The eight most significantly down-regulated genes with introduction of the LH receptor, and affected pathways. Table S6 Pathways impacted by LHR introduction (fold-change of gene expressed indicated in parentheses). Table S7 Pathways impacted by LH treatment (fold-change of gene expressed indicated in parentheses). Table S8 Expression differentiation of the genes involved in the MAPK pathway. Table S9 Two lists of genes including 185 and 248, respectively, which are identified as highly-expressed and under-expressed in all cancer cells versus normal cells. (The 106 genes that are specific to ovarian cancer and 103 that are predicted to be blood secreted are indicated). Table S10 48 therapeutic targets reported to be ovarian cancer-associated (expression fold-changes are shown; "-" indicates that the corresponding genes are not included in the microarray chip platform). Table S11 Differential gene expression compared to those observed in other cells modulated by LH or FSH. [file 1471-2407-11-280-S1.DOC]

**Table S1** 23 differentially expressed genes that are used for PCR validation

<http://csbl.bmb.uga.edu/~juancui/Publications/OvCan2011/Supplementary_table_1_genes_for_PCR.xls>

**Table S2** Statistics of genes with their geometric mean fold-change >=2 across any time course.

|  | **LHR+** | **LH1h** | **LH4h** | | **LH8h** | | **LH20h** | |
| --- | --- | --- | --- | --- | --- | --- | --- | --- |
|  | *LHR+/LHR-* | *LH1/LHR+* | *LH4/LHR+* | *LH4/LH1* | *LH8/LHR+* | *LH8/LH4* | *LH20/LHR+* | *LH20/LH8* |
| **Up** | 144 | 137 | 296 | 191 | 182 | 108 | 665 | 410 |
| **Down** | 270 | 13 | 167 | 178 | 130 | 199 | 708 | 441 |
| **Total** | 414 | 150 | 463 | 369 | 312 | 307 | 1373 | 851 |
| unique genes involved: 2,373 | | | | | | | | |
| Cancer related genes: 689 ; Ovarian expressed: 265 | | | | | | | | |

**Table S3** Identified 2373 differentially expressed genes, among which 689 are reported to be cancer related.

<http://csbl.bmb.uga.edu/~juancui/Publications/OvCan2011/Supplementary_table_3_identified_genes.xls>

**Table S4** 12 expression patterns that identified in 2373 differentially expression genes, which is corresponding to the blue plots in Table 1 in the main text. The detailed gene lists for each clusters can be accessed at <http://csbl.bmb.uga.edu/~juancui/Publications/OvCan2011/Supplementary_table_4_identified_clusters.xls>

**Table S5** The eight most significantly down-regulated genes with introduction of the LH receptor, and affected pathways

| **Gene** | **Max. fold Change with LHR** | **Pathways in which the gene is involved** |
| --- | --- | --- |
| CFI | -46 | Complement and coagulation cascades |
| ERBB4 | -46 | ErbB signaling pathway  Calcium signaling pathway  Dorso-ventral axis formation |
| CASP1 | -44 | Neurodegenerative Diseases  Huntington's disease  Dentatorubropallidoluysian atrophy (DRPLA) |
| UGT8 | -43 | Sphingolipid metabolism |
| KRT16 | -42 | Cell Communication |
| PLN | -42 | Calcium signaling pathway |
| TYR | -38 | Tyrosine metabolism  Riboflavin metabolism  Alkaloid biosynthesis I  Melanogenesis |
| COL3A1 | -37 | Cell Communication  Focal adhesion  ECM-receptor interaction |

**Table S6** Pathways impacted by LHR introduction (fold-change of gene expressed indicated in parentheses)

| **Pathway** | **General function** | **Differentially expressed genes with LHR introduction** | | | **LH1/LHR+** | **LH4/LHR+** | **LH8/LHR+** | **LH20/LHR+** |
| --- | --- | --- | --- | --- | --- | --- | --- | --- |
|  |  | **Genes involved** | **Up**  **regulated** | **Down**  **regulated** |  |  |  |  |
| Cell communication | Cell growth, signal transmitting, including Focal adhesion, adherens junction, tight junction, gap junction | COL11A1  DSC3  LAMB3  KRT7  COL4A1  KRT17  LAMC2  COL6A3  LAMA3  COL5A2  TNC  COL1A2  COL3A1  KRT16 | COL11A1 (52) | DSC3 (-2)  LAMB3 (-2)  KRT7 (-2)  COL4A1 (-2)  KRT17 (-3)  LAMC2 (-3)  COL6A3 (-4)  LAMA3 (-5)  COL5A2 (-5)  TNC (-5)  COL1A2 (-8)  COL3A1 (-37)  KRT16 (-42) |  |  | KRT17 (3) | LAMA3 (2)  COL5A2 (3)  TNC (-2)  COL1A2 (3) |
| ECM-receptor interaction | tissue and organ morphogenesis; maintenance of cell and tissue structure and function; control of cellular activities such as adhesion, migration, differentiation, proliferation, and apoptosis | COL11A1  LAMB3  COL4A1  LAMC2  COL6A3  LAMA3  TNC  ITGA11  COL1A2  COL3A1  COL5A2 | COL11A1 (52) | LAMB3 (-2)  COL4A1 (-2)  LAMC2 (-3)  COL6A3 (-4)  LAMA3 (-5)  TNC (-5)  ITGA11 (-7)  COL1A2 (-8)  COL3A1 (-37)  COL5A2 (-5) |  |  |  | LAMA3 (2)  TNC (-2)  COL1A2 (3)  COL5A2 (3) |
| Focal adhesion | cell motility, cell proliferation, cell differentiation, regulation of gene expression and cell survival | COL11A1  VAV3  LAMB3  COL4A1  LAMC2  PIK3R3  COL6A3  LAMA3  PAK6  TNC  ITGA11  COL1A2  COL3A1  COL5A2 | COL11A1 (52)  VAV3 (7) | LAMB3 (-2)  COL4A1 (-2)  LAMC2 (-3)  PIK3R3 (-3)  COL6A3 (-4)  LAMA3 (-5)  PAK6 (-5)  TNC (-5)  ITGA11 (-7)  COL1A2 (-8)  COL3A1 (-37)  COL5A2 (-5) |  | --  VAV3 (-3) |  | --  VAV3 (6)  LAMA3 (2)  TNC (-2)  COL1A2 (3)  COL5A2 (3) |
| Eicosanoid metabolism | intracellular signal cascades; complex control in [inflammation](http://en.wikipedia.org/wiki/Inflammation) or [immunity](http://en.wikipedia.org/wiki/Immune_system) | CYP2J2  PTGIS  PTGS1 | PTGS1 (3) | CYP2J2 (-3)  PTGIS (-5) |  |  |  |  |
| IL 18 Signaling pathway | stimulates cell differentiation and involvement in immune responses; induction of apoptosis | IL18  CASP1 |  | IL18 (-2)  CASP1 (-44) |  |  |  |  |
| Complement and coagulation cascade | opsonization of pathogens, the recruitment of inflammatory and immunocompetent cells, and the direct killing of pathogens | PLAUR  CD55  PLAT  SERPINA1  C1S  CFH  CFI |  | PLAUR (-2)  CD55 (-3)  PLAT (-3)  SERPINA1 (-4)  C1S (-5)  CFH (-19)  CFI (-46) |  | PLAUR (2)  CD55 (3) | CD55 (3) | CD55 (4)  PLAT (3)  SERPINA1 (-3)  CFH (2) |
| Nitrogen metabolism |  | CA2  ASRGL1  GLS | CA2 (3)  ASRGL1 (3) | GLS (-2) |  |  | GLS (2) | CA2 (-2)  ASRGL1 (-3)  GLS(3) |
| Notch signaling pathway | intercellular signaling mechanism essential for proper embryonic development | DTX1  JAG2  JAG1  HES1 | DTX1 (7)  JAG2 (4) | JAG1 (-2)  HES1 (-4) | HES1 (4) | JAG1 (2) |  | HES1 (2) |
| Nicotinate and nicotinamide metabolism |  | NNT  BST1  NMNAT2 | NNT (13)  BST1 (5) | NMNAT2 (-2) |  |  |  | NMNAT2 (-2) |
| ErbB signaling pathway | regulate proliferation, differentiation, cell motility, and survival | NRG1  PIK3R3  BTC  PAK6  ERBB4 | NRG1 (4) | PIK3R3 (-3)  BTC (-5)  PAK6 (-5)  ERBB4 (-46) |  |  |  | NRG1 (-8)  ERBB4 (6) |
| Small cell lung cancer | cell-cycle progression; proliferation and apoptosis | LAMB3  COL4A1  LAMC2  PIK3R3  LAMA3 |  | LAMB3 (-2)  COL4A1 (-2)  LAMC2 (-3)  PIK3R3 (-3)  LAMA3 (-5) |  |  |  | LAMA3 (2) |
| Leukocyte transendothelial migration | immune surveillance and inflammation | VAV3  CLDN4  MMP2  RAPGEF3  F11R  PIK3R3 | VAV3 (7) | CLDN4 (-2)  MMP2 (-2)  RAPGEF3 (-2)  F11R (-3)  PIK3R3 (-3) |  | VAV3 (-3)  CLDN4 (-2) |  | VAV3 (6)  RAPGEF3 (2)  F11R (2) |
| TGF-beta signaling pathway | proliferation, apoptosis, differentiation and migration regulation | INHBB  BMP8B  BAMBI  CUL1  INHBE  DCN | INHBB (6)  BMP8B (3) | BAMBI (-2)  CUL1 (-4)  INHBE (-4)  DCN (-5) |  | INHBE (-3) |  | BAMBI (-4)  CUL1 (-3)  INHBE (-5)  DCN (7) |
| Axon guidance | key stage in the formation of neuronal network | SLIT3  SEMA4G  ABLIM3  NTNG1  PLXNA2  PAK6 | SLIT3 (5)  SEMA4G (4) | ABLIM3 (-2)  NTNG1 (-3)  PLXNA2 (-4)  PAK6 (-5) |  |  |  | SEMA4G (2)  NTNG1 (2) |
| Calcium signaling pathway | signal transduction, e.g. involved in muscle contraction | BST1  ADORA2B  PDE1C  ADRB2  CCKAR  PLN  ERBB4 | BST1 (5)  ADORA2B (4)  PDE1C (3) | ADRB2 (-2)  CCKAR (-17)  PLN (-42)  ERBB4 (-46) | ADRB2 (4)  PLN (2) |  | ADORA2B (-2) | ADORA2B (-3)  ERBB4 (6) |
| Cytokine-cytokine receptor interaction | intercellular regulation of innate as well as adaptive inflammatory host defenses, cell growth, differentiation, cell death, angiogenesis, and development and repair processes | INHBB  IL18  IL1R1  FAS  KITLG  INHBE  TNFRSF19  CXCL1  TNFSF10 | INHBB (6) | IL18 (-2)  IL1R1 (-3)  FAS (-3)  KITLG (-4)  INHBE (-4)  TNFRSF19 (-5)  CXCL1 (-5)  TNFSF10 (-7) | CXCL1 (8) | IL1R1 (4)  INHBE (-3)  CXCL1 (6) | IL1R1 (3) | IL1R1 (4)  INHBE (-5)  CXCL1 (5)  TNFSF10 (69) |
| Apoptosis | cell death | IL1R1  FAS  PIK3R3  TNFSF10 |  | IL1R1 (-3)  FAS (-3)  PIK3R3 (-3)  TNFSF10 (-7) |  | IL1R1 (4) | IL1R1 (3) | IL1R1 (4)  TNFSF10 (69) |
| Acute myeloid leukemia | increased proliferation and apoptosis resistance | PIK3R3  CCNA1  JUP |  | PIK3R3 (-3)  CCNA1 (-5)  JUP (-7) |  |  |  | JUP (3) |
| Arachidonic acid metabolism |  | PTGS1  CYP2J2  PTGIS | PTGS1 (3) | CYP2J2 (-3)  PTGIS (-5) |  |  |  |  |
| Natural killer cell mediated cytotoxicity | Immune response, induce cell death | VAV3  ULBP2  FAS  PIK3R3  TNFSF10 | VAV3 (7) | ULBP2 (-3)  FAS (-3)  PIK3R3 (-3)  TNFSF10 (-7) |  | VAV3 (-3) |  | VAV3 (6)  TNFSF10 (69) |
| P53 signaling pathway | cell cycle arrest, cellular senescence or apoptosis | GTSE1  FAS  SERPINB5  TIMP3 |  | GTSE1 (-2)  FAS (-3)  SERPINB5 (-8)  TIMP3 (-20) |  | GTSE1 (-3) |  | GTSE1 (-2) |
| Purine metabolism |  | NME4  GUCY1B3  PDE1C  PDE5A  GDA | NME4 (5)  GUCY1B3 (3)  PDE1C (3) | PDE5A (-2)  GDA (-6) |  |  | PDE5A (-3) | GUCY1B3 (3) |
| Hematopoietic cell lineage | Blood-cell development | CD55  IL1R1  KITLG |  | CD55 (-2)  IL1R1 (-3)  KITLG (-4) |  | CD55 (3)  IL1R1 (4) | CD55 (3)  IL1R1 (3) | CD55 (4)  IL1R1 (4) |
| Regulation of actin cytoskeleton |  | VAV3  FGF12  PIK3R3  PAK6  ITGA11  FGFR2 | VAV3 (7)  FGF12 (4)  FGFR2 (4) | PIK3R3 (-3)  PAK6 (-5)  ITGA11 (-7) | FGFR2 (4) | VAV3 (-3)  FGFR2 (2) | FGFR2 (2) | VAV3 (6)  FGFR2 (11) |
| MAPK signaling pathway | cell proliferation, differentiation and migration | CACNG4  RASGRF2  FGF12  FGFR2  CACNA2D3  IL1R1  FAS | CACNG4 (7)  RASGRF2 (7)  FGF12 (4)  FGFR2 (4) | CACNA2D3(-2)  IL1R1 (-3)  FAS (-3) | FGFR2 (4) | FGFR2 (2)  IL1R1(4) | FGFR2 (2)  IL1R1 (3) | FGFR2 (11)  CACNA2D3(3)  IL1R1 (4) |
| T cell receptor signaling pathway | Immune response, leading to T-cell proliferation, cytokine production and differentiation into effector cells | VAV3  CAMK1  PIK3R3  PAK6 | VAV3 (7)  CAMK1 (6) | PIK3R3 (-3)  PAK6 (-5) |  | VAV3 (-3) |  | VAV3 (6) |
| Gap junction | essential embryonic development, electrical coupling, metabolic transport, apoptosis, and tissue homeostasis | TUBAL3  TUBB2B  GUCY1B3 | TUBAL3 (6)  TUBB2B (4)  GUCY1B3 (3) |  |  |  |  | TUBAL3 (-2)  GUCY1B3 (3) |
| Cell cycle |  | BUB1  CUL1  CCNA1 |  | BUB1 (-2)  CUL1 (-4)  CCNA1 (-5) |  | BUB1 (-3) | BUB1 (-2) | BUB1 (-3)  CUL1 (-3) |
| Glycan structures- biosynthesis 1 |  | GALNT7  GALNT3  NDST4 |  | GALNT7 (-2)  GALNT3 (-4)  NDST4 (-23) |  |  | GALNT3 (2) | GALNT3 (3) |
| Tight junction | mediate cell adhesion and constitute the intramembrane and paracellular diffusion barriers; establishment and maintenance of epithelial cell polarity; barrier regulation and gene transcription | RAB3B  F11R  CLDN4 | RAB3B (6) | F11R (-3)  CLDN4 (-2) |  | CLDN4 |  | F11R (2) |
| Neuroactive ligand-receptor interaction |  | ADORA2B  PRSS1  NMNAT2  F2RL2  ADRB2  CCKAR | ADORA2B (4) | PRSS1 (-2)  NMNAT2 (-2)  F2RL2 (-2)  ADRB2 (-2)  CCKAR (-17) | ADRB2 (4) |  | ADORA2B (-2) | ADORA2B (-3)  NMNAT2 (-2) |

**Table S7** Pathways impacted by LH treatment (fold-change of gene expressed indicated in parentheses)

| **Pathway** | **General function** | **Genes involved** | **LH1/LHR+** | **LH4/LHR+** | **LH8/LHR+** | **LH20/LHR+** |
| --- | --- | --- | --- | --- | --- | --- |
| MapKinase Signaling Pathway | Gene expression, mitosis, cell survival/apoptosis, differentiation | FOS, DUSP5, DUSP1, NR4A1, MYC, BDNF, DUSP6, MAPK1, FGFR2 | FOS (16)  MYC (3)  DUSP5 (5)  BDNF (3)  DUSP1 (5)  DUSP6 (2)  NR4A1 (4)  FGFR2 (4)  MAPK1 (-2) | DUSP5 (5)  BDNF (5)  NR4A1 (4)  FGFR2 (2) | BDNF (2)  DUSP6 (-2)  FGFR2 (2) | BDNF(3)  DUSP1 (2)  FGFR2(11) |
| Erk1/Erk2 Mapk Signaling pathway | growth control and development, underlying normal and malignant cell proliferation and differentiation | MYC, MAPK1, ITGB1 | MYC (3)  ITGB1 (4)  MAPK1 (-2) |  |  |  |
| p38 MAPK Signaling Pathway | cell differentiation and [apoptosis](http://en.wikipedia.org/wiki/Apoptosis); responsive to stress stimuli, such as [cytokines](http://en.wikipedia.org/wiki/Cytokines), | MYC | MYC (3) |  |  |  |
| Cytokine-cytokine receptor interaction | Cellular communication, development and functioning of both the innate and adaptive immune response | CCL20, CXCL2, CXCL3, IL8, C7orf44, INHBA, PF4V1, CXCL1 | CCL20(44) CXCL1 (8)  CXCL2(11) PF4V1 (2)  C7orf44 (2)  IL8 (7)  CXCL3 (7) INHBA (2) | CCL20 (33)  CXCL1 (6)  CXCL2 (6)  PF4V1 (5)  IL8 (5)  CXCL3 (5)  INHBA (3) | --  --  --  PF4V1 (4)  --  --  --  -- | --  CXCL1 (5)  CXCL2 (2)  PF4V1 (8)  --  --  --  INHBA (-4) |
| TGF-beta signaling pathway | Cell growth, cell differentiation, apoptosis, cell homeostasis | THBS1, MYC, ID3, INHBA, MAPK1 | THBS1 (4)  ID3 (2)  MYC (3) INHBA(2)  MAPK (-2) | THBS1 (2)  ID3 (6)  INHBA (3) | THBS1 (5) ID3 (3)  --  -- | --  ID3 (11)  --  INHBA (-4) |
| ErbB signaling pathway | proliferation, migration, differentiation, and survival or apoptosis. neuronal migration and differentiation | MYC, HBEGF, MAPK1 | HBEGF (3)  MAPK (-2) | HBEGF (4) | --  -- | --  -- |
| GnRH signaling pathway | FSH and LH regulation | HBGEF, MAPK1, GNRH1 | HBEGF (3)  MAPK (-2)  GNRH1 (-2) | HBEGF (4) | -- | -- |
| Toll-like receptor signaling pathway | Key in innate immune system, cytokine production and cellular activation in response to microbes; innate and adaptive immune responses, apoptosis, proinflammatory effect, t cell stimulation, antiviral effects, proinflammatory effect | FOS, IL8, MAPK1 | IL8 (7)  FOS (16)  MAPK (-2) | IL8 (5) | -- | -- |
| Focal adhesion | Cell motility, cell cycle, immune system, cytoskeleton, regulate signaling complexes and integrin function, cell proliferation, survival, migration, and invasion. | THBS1, ITGB1, CTNNB1, MAPK1 | THBS1 (4)  ITGB1 (4)  CTNNB1(2)  MAPK (-2) | THBS1 (2)  CTNNB1 (2) | THBS1 (5) | -- |
| Regulation of actin cytoskeleton | regulation of cell shape, motility, and adhesion | ITGB1, SSH1, MAPK1, FGFR2 | ITGB1 (4)  SSH1 (2)  FGFR2 (4)  MAPK (-2) | SSH1 (2)  FGFR2 (2) | FGFR2 (2) | FGFR2 (11) |
| Jak-STAT signaling pathway | regulation of cellular responses to [cytokines](http://en.wikipedia.org/wiki/Cytokine) and [growth factors](http://en.wikipedia.org/wiki/Growth_factor). Regulating the processes of [cell proliferation](http://en.wikipedia.org/wiki/Cell_proliferation), [differentiation](http://en.wikipedia.org/wiki/Cell_differentiation) and [apoptosis](http://en.wikipedia.org/wiki/Apoptosis). It is particularly important in [hematopoiesis](http://en.wikipedia.org/wiki/Hematopoiesis) - production of [blood cells](http://en.wikipedia.org/wiki/Blood_cell). | MYC, SPRY2, SPRY4 | MYC (3)  SPRY2 (3)  SPRY4 (2) | -- | -- | -- |
| T cell receptor signaling pathway | Immune system, T-cell proliferation, cytokine production and differentiation into effector cells, immune response, regulation of actin cytoskeleton, ubiquitin mediated proteolysis | FOS, BCL10 | FOS (16)  BCL10 (2) |  |  |  |
| B cell receptor signaling pathway | differentiation, survival, apoptosis, proliferation and tolerance. | FOS, BCL10 | FOS (16)  BCL10 (2) |  |  |  |
| Cadmium induced DNA synthesis and proliferation in macrophages | In addition to inducing cellular proliferation, cadmium also is slightly genotoxic due to inhibition of DNA repair, activates stress genes, and inhibits the immune system. Reduced phagocytic activity | FOS, MYC, MAPK1 | FOS (16)  MYC (3)  MAPK (-2) |  |  |  |
| Role of EGF Receptor Transactivation by GPCRs in Cardiac Hypertrophy |  | FOS, EDN1, MYC | FOS (16)  MYC (3)  EDN1 (10) | EDN1 (3) | --  --  EDN1 (2) | --  --  EDN1(2) |
| Neuropeptides VIP and PACAP inhibit the apoptosis of activated T cells |  | EGR2, EGR3, MYC | MYC (3)  EGR2 (8)  EGR3 (6) |  |  |  |
| Oxidative Stress Induced Gene Expression Via Nrf2 | Enzymes are involved in the Phase II detoxification of xenobiotics to reduce cellular stress. Expression of these genes protects cells from oxidative damage and can prevent mutagenesis and cancer. Transcription of these enzymes is coordinately regulated through antioxidant response elements (AREs). Nrf2 (NF-E2-related factor 2) and Nrf1 are transcription factors that bind to AREs and activate these genes. | FOS, MAFF, MAPK1 | FOS (16)  MAFF (2)  MAPK (-2) | MAFF (3) | --  -- | --  -- |
| NFAT and Hypertrophy of the heart |  | HBGEF, MAPK1, EDN1 | HBEGF (3)  EDN1 (10)  MAPK (-2) | HBEGF (4)  EDN1 (3) | EDN1 (2) | EDN1 (2) |
| Pathogenic Escherichia coli infection - EPEC | Inflammation, cytoskeleton rearrangment, regulation of actin cytoskeleton, apoptosis, disruption of tight and adherens junction, disruption of barrier function, increase in monolayer permeability, inhibition of protein synthesis | ITGB1, CTNNB1, ARHGEF2 | ITGB1 (4)  CTNNB1 (2)  ARHGEF2 (-2) | CTNNB1 (2) | --  -- | --  -- |
| Pathogenic Escherichia coli infection - EHEC |  |  |  |  |  |  |
| Chemokine pathway | induced during an immune response to promote cells of the [immune system](http://en.wikipedia.org/wiki/Immune_system) to a site of [infection](http://en.wikipedia.org/wiki/Infection), while others are considered [homeostatic](http://en.wikipedia.org/wiki/Homeostatic) and are involved in controlling the migration of cells during normal processes of tissue maintenance or [development](http://en.wikipedia.org/wiki/Developmental_biology). Some chemokines have roles in development; they promote [angiogenesis](http://en.wikipedia.org/wiki/Angiogenesis) (the growth of new [blood vessels](http://en.wikipedia.org/wiki/Blood_vessel)), or guide cells to tissues that provide specific signals critical for cellular maturation. | CXCL1,, CXCL2, CXCL3 | CXCL1 (8)  CXCL2 (11)  CXCL3 (7) | CXCL1 (6)  CXCL2 (6)  CXCL3 (5) | --  --  -- | CXCL1 (5)  CXCL2 (2)  -- |
| Epithelial cell signaling in Helicobacter pylori infection | Apical junctional complex disruption, cell motility, proliferation, apoptosis, chemotactic effect, proinflammatory effect, vacuolation | IL8, HBEGF, CXCL1 | CXCL1 (8)  HBEGF (3)  IL8 (7) | CXCL1(6)  HBEGF (4)  IL8 (5) | --  --  -- | CXCL1 (5)  --  -- |
| Melanogenesis | cells produce [melanin](http://en.wikipedia.org/wiki/Melanin), which is a pigment found in the [skin](http://en.wikipedia.org/wiki/Skin), [eyes](http://en.wikipedia.org/wiki/Eye), and [hair](http://en.wikipedia.org/wiki/Hair); Ultraviolet rays penetrate the skin and damage DNA; thymidine dinucleotides (pTpT) fragments from damaged DNA will trigger release of the hormone alpha-MSH, which can then bind to melanocytes to cause them produce melanin. | EDN1, CREB3L2, CTNNB1, MAPK1 | CTNNB1 (2)  EDN1 (10)  CREB3L2(2)  MAPK (-2) | CTNNB1 (2)  EDN1 (3)  CREB3L2(3) | --  EDN1 (2)  CREB3L2 (3) | --  EDN1 (2)  CREB3L2(3) |
| Trefoil Factors Initiate Mucosal Healing | epithelial cells spread and migrate across the basement membrane to re-establish surface-cell continuity, a process that is independent of cell proliferation. Epithelial continuity depends on a family of small abundant secreted proteins, the trefoil factors (TFFs). TFF response elements in TFF gene promoters allow increases in TFF expression through auto-induction and cross-induction of other TFFs, in addition to mucin expression and possibly tumor suppression. | ITGB1, MAPK1, CTNNB1 | ITGB1 (4)  CTNNB1 (2)  MAPK (-2) | CTNNB1 (2) | --  -- | --  -- |
| Thyroid cancer | Proliferation, survival, genomic instability, impared G1 cycle arrest, reduced apoptosis | MYC, CTNNB1, MAPK1 | CTNNB1(2)  MYC (3)  MAPK (-2) | CTNNB1 (2) | -- | -- |
| Colorectal cancer | Proliferation, anti-apoptosis, loss of growth inhibitory effects of TGF beta, karyotypic instability impaired G1 cycle arrest reduced apoptosis, survival, suppressed apoptosis, cytoskeleton remodeling | FOS, MYC, CTNNB1, MAPK1 | CTNNB1 (2)  MYC (3)  FOS (16)  MAPK (-2) | CTNNB1 (2) | -- | -- |
| Endometrial cancer | angiogenesis, cell growth and proliferation and chromosomal instability, impaired G1 and G2 arrest, reduced apoptosis, genomic instability | MYC, CTNNB1, MAPK1 | CTNNB1(2)  MYC (3)  MAPK (-2) | CTNNB1 (2) |  |  |
| Bladder cancer | Angiogenesis, G1/S progression, cell cycle | IL8, THBS1, MYC, MAPK1 | IL8 (7)  MYC (3)  THBS1 (4)  MAPK (-2) | IL8 (5) | -- | -- |
| Prostate cancer | G1/S progression, cell cycle, impaired G1 and G2 arrest, reduced apoptosis, genomic instability, cell proliferation, cell survival | CREB3L2, CTNNB1, FGFR2, DLC1 | CTNNB1(2)  CREB3L2(2)  FGFR2 (4)  DLC1 (3) | CTNNB1 (2)  CREB3L2(3)  FGFR2 (2) | CREB3L2(3)  FGFR2 (2)  -- | CREB3L2(3)  FGFR2 (11)  -- |

**Table S8** Expression differentiation of the genes involved in the MAPK pathway

|  | **LHR+/LHR-** | **LH1/LHR+** | **LH4/LHR+** | **LH8/LHR+** | **LH20/LHR+** |
| --- | --- | --- | --- | --- | --- |
| **Up regulated** | CACNG4 (7)  RASGRF2 (7)  FGF12 (4)  FGFR2 (4)  DNM1 (3) | FOS (16)  MYC (3)  DUSP5 (5)  BDNF (3)  DUSP1 (5)  DUSP6 (2)  NR4A1 (4)  FGFR2 (4)  ITGB1 (4) | DUSP5 (5)  BDNF (5)  NR4A1 (4)  FGFR2 (2)  IL1R1(4)  KRAS (2)  NFATC2 (6)  IGF1R (2)  CEBPA (2) | BDNF (2)  FGFR2 (2)  IL1R1 (3)  PTPRR (2)  NFATC2 (4)  IGF1R (2) | BDNF(3)  DUSP1 (2)  FGFR2(11)  CACNA2D3 (3)  IL1R1 (4)  PTPRR (6)  RPS6KA5 (2)  PLA2G6 (3)  MAP3K12 (3)  MDS1 (9)  PDGFRA (4)  FGF11 (3)  MAP2K6 (16)  MAPK8IP3 (2) |
| **Down regulated** | CACNA2D3 (-2)  IL1R1 (-3)  FAS (-3) | MAPK1 (-2) | MAP3K14 (-2)  NGFB (-3)  PDGFB (-2)  MAP3K14 (-2) | DUSP6 (-2)  CEBPA (-2)  CEBPA (-2) | NGFB (-4)  PDGFB (-2)  DUSP4 (-2)  FGF7 (-3)  NF1 (-2)  GADD45A (-3)  JUN (-2)  MAX (-2)  PPP3CB (-3)  CACNA1G (-3)  FGF2 (-3)  PAK2 (-2) |

**Table S9** Two lists of genes including 185 and 248, respectively, which are identified as highly-expressed and under-expressed in all cancer cells versus normal cells. (The 106 genes that are specific to ovarian cancer and 103 that are predicted to be blood secreted are indicated)

<http://csbl.bmb.uga.edu/~juancui/Publications/OvCan2011/Supplementary_table_9_HOSEvsSKOV3.xls>

**Table S10** 48 therapeutic targets reported to be ovarian cancer-associated (expression fold-changes are shown; “-“ indicates that the corresponding genes are not included in the microarray chip platform)

| **Type** | **Target name** | **Symbol** | **Expression differentiation (fold change)** | | | | |
| --- | --- | --- | --- | --- | --- | --- | --- |
|  |  |  | **LHR+** | **LH1** | **LH4** | **LH8** | **LH20** |
| **S**uccessful  target | Vascular endothelial growth factor receptor 1 | FLT1 | -1.3 | 1.7 | -1.1 | -1.0 | -1.1 |
|  | Vascular endothelial growth factor | VEGFA | -1.0 | 1.0 | -1.2 | -1.1 | -1.2 |
|  | Tubulin | TUB | 1.2 | 1.3 | 2.3 | 1.3 | -1.4 |
|  | Thymidylate synthase | TYMS | 1.2 | 1.3 | 1.8 | 1.3 | -1.1 |
|  | Receptor protein-tyrosine kinase erbB-2 | ERBB2 | 1.4 | -1.1 | 1.3 | 1.2 | 2.0 |
|  | Proto-oncogene tyrosine-protein kinase receptor | RET | 1.7 | 1.8 | 2.8 | 2.6 | 1.6 |
|  | Poly [ADP-ribose] polymerase-1 | PARP1 | -1.0 | -1.0 | -1.0 | -1.1 | -1.0 |
|  | Multidrug resistance-associated protein 1 | ABCC1 | -1.0 | 1.0 | -1.0 | -1.1 | 1.0 |
|  | Multidrug resistance protein | ABCC1 | -1.0 | 1.0 | -1.0 | -1.1 | 1.0 |
|  | Vascular endothelial growth factor receptor 2 | VEGFR-2 | - | - | - | - | - |
|  | Mast/stem cell growth factor receptor | KIT | 1.4 | 1.2 | -1.0 | 2.9 | 3.1 |
|  | Gonadotropin-releasing hormone receptor | GNRHR | -1.8 | 3.2 | 1.5 | 2.0 | 3.0 |
|  | tGastrin/cholecystokinin type B receptor | CCKBR | 2.3 | -3.2 | -2.4 | -14.3 | -4.9 |
|  | Epidermal growth factor receptor | EGFR | 1.1 | 1.1 | -1.1 | -1.4 | -1.3 |
|  | Endothelin-1 receptor | EDN1 | -1.1 | 10.2 | 4.3 | 1.8 | 2.1 |
|  | DNA topoisomerase II | TOP2A | -1.2 | -1.6 | -1.9 | -2.0 | -2.3 |
|  | DNA topoisomerase I | TOP1 | 1.0 | 1.0 | 1.1 | -1.3 | -1.3 |
|  | B-Raf proto-oncogene serine/threonine-protein kinase | BRAF | 1.1 | 1.3 | 1.1 | 1.1 | -2.4 |
| **C**linical  target | Ubiquitin-protein ligase E3 Mdm2 | MDM2 | 1.7 | -1.1 | -1.2 | -1.0 | -3.9 |
|  | Transforming growth factor-beta | GFB | - | - | - | - | - |
|  | Stromelysin-1 | MMP3 | -1.4 | -1.7 | 1.3 | 1.8 | 1.7 |
|  | Sphingosine kinase | SPHK | - | - | - | - | - |
|  | Serine/threonine-protein kinase 6 | STK6 | - | - | - | - | - |
|  | RAC-alpha serine/threonine kinase | AKT1 | 1.0 | -1.0 | 1.1 | 1.1 | -1.0 |
|  | Poly [ADP-ribose] polymerase 1 | PARP1 | -1.0 | -1.0 | -1.0 | -1.1 | -1.0 |
|  | Phosphatidylinositol-4,5-bisphosphate 3-kinase catalytic subunit, gamma isoform | PIK3CA | -1.5 | 1.2 | 1.5 | 1.2 | 2.0 |
|  | Neural-cadherin | CDH | - | - | - | - | - |
|  | mRNA of Inhibitor of apoptosis protein | API | - | - | - | - | - |
|  | mRNA of Heat shock 27 kDa protein | HSPB1 | -1.1 | 1.1 | 1.1 | 1.3 | 1.7 |
|  | Kinesin-like protein KIF11 | KIF11 | -1.5 | -1.1 | -2.3 | -1.3 | -1.6 |
|  | Interleukin-18 | IL18 | -2.1 | 1.1 | 1.2 | -1.0 | -1.2 |
|  | Histone deacetylase 4 | HDAC4 | 1.3 | -1.1 | 1.0 | 1.1 | -1.1 |
|  | Histone deacetylase | HDAC | - | - | - | - | - |
|  | Heat shock protein HSP 90 | HSP90A | - | - | - | - | - |
|  | Folate receptor alpha | FOLR1 | 1.6 | 1.7 | 1.3 | -1.1 | 1.1 |
|  | C-X-C chemokine receptor type 4 | CCR4 | 3.1 | -1.3 | -1.6 | -2.1 | -1.8 |
|  | C-C chemokine receptor type 1 | CCR1 | -1.4 | 2.1 | 1.3 | 2.2 | 2.7 |
|  | Vascular endothelial growth factor receptor 3 | FLT4 | -1.3 | 1.3 | 2.0 | 1.5 | 1.2 |
| **R**esearch  target | Stromal cell-derived factor 1 | CXCL12 | 1.2 | 1.6 | 10.3 | 6.3 | 4.2 |
|  | P185HER-2 | ERBB2 | 1.4 | -1.1 | 1.3 | 1.2 | 2.0 |
|  | Neuropsin | KLK8 | 2.1 | -1.1 | -1.2 | -1.2 | -1.1 |
|  | Neuropeptide Y receptor | NPY | -2.3 | 1.8 | 1.2 | 1.2 | 1.3 |
|  | Mitogen-activated protein kinase 4 | MPK4 | - | - | - | - | - |
|  | Kallikrein 7 | KLK7 | -1.1 | -1.4 | -1.2 | 1.2 | 1.9 |
|  | Insulin-like growth factor II | IGF2 | -8.7 | 8.6 | 11.6 | 3.9 | 9.2 |
|  | Focal adhesion kinase | PTK | - | - | - | - | - |
|  | Fascin | FSCN1 | 1.3 | 1.2 | 1.1 | 1.1 | 1.5 |
|  | Fanconi anemia group F protein | FANCA | 1.1 | -1.2 | -1.3 | -1.6 | -2.0 |
|  | DNA-(apurinic or apyrimidinic site) lyase | APEX1 | 1.1 | -1.1 | 1.1 | 1.1 | 1.1 |
|  | Breast cancer type 2 susceptibility protein | BRCA2 | -1.3 | 1.5 | -1.0 | -1.2 | -1.4 |

**Table S11** Differential gene expression compared to those observed in other cells modulated by LH or FSH.

<http://csbl.bmb.uga.edu/~juancui/Publications/OvCan2011/Supplementary_table_11_otherCelltype.xls>
