# Supplementary material for: Regulation of gene expression in ovarian cancer cells by luteinizing hormone receptor expression and activation
Source: BMC Cancer. 2011 Jun 28;11:280. doi: 10.1186/1471-2407-11-280 (PMC3141782; doi:10.1186/1471-2407-11-280)
Supplement: Additional file 2 — Supplementary figures. Figure S1 Evaluation of the Microarray data quality and. Figure S2 qRT-PCR measures of gene TNFSF10 and ET-1 [file 1471-2407-11-280-S2.DOC]

**Figure S1** Evaluation of the Microarray data quality A) The hierarchical clustering shows the grouping results as expected based on Euclidean distance with transcripts passing 3SD Bg filter. The six sample groups are control (ctrl) and 0, 1, 4, 8 and 20 hours treatment (0_hr, 1_hr, 4_hrs, 8_hrs and 20_hrs).


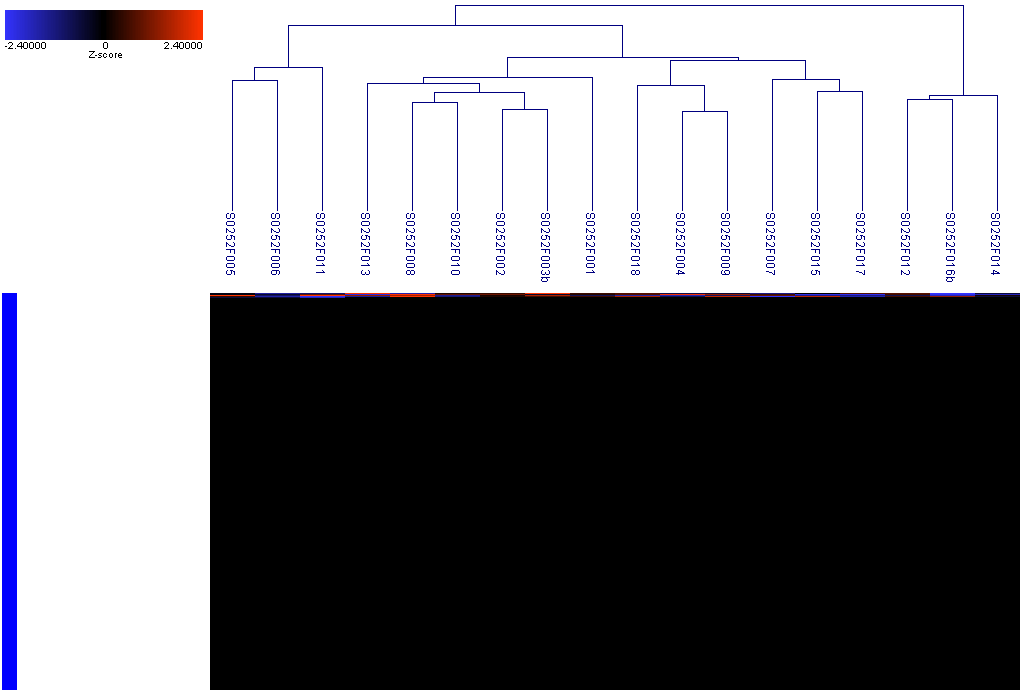


**ctrl** **0_hr 1_hr 4_hrs 8_hrs 20_hrs**

B).PCA result shows sample relationships grouping in a manner consistent with the study design. The PCA plot was generated with transcripts passing 3SD Bg filter.


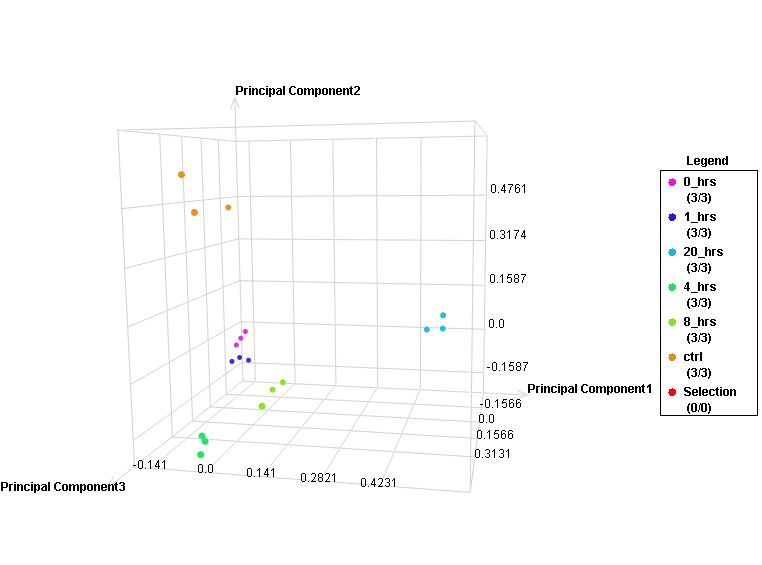


**Figure S2 A)** qRT-PCR measures of gene TNFSF10 (top panel; primer sequence (F) AAGACTGTCAGCTTCCAAACATTAA (R) GTGATACACTACTTGAGAGATGGAT) **and B)** ET-1 (lower panel; primer sequence (F) AGGCCCTGAGTTGGCAGTGGCCCAT(R)ATGGGCCACTGCCAACTCAGGGCCT)

**A:**

**
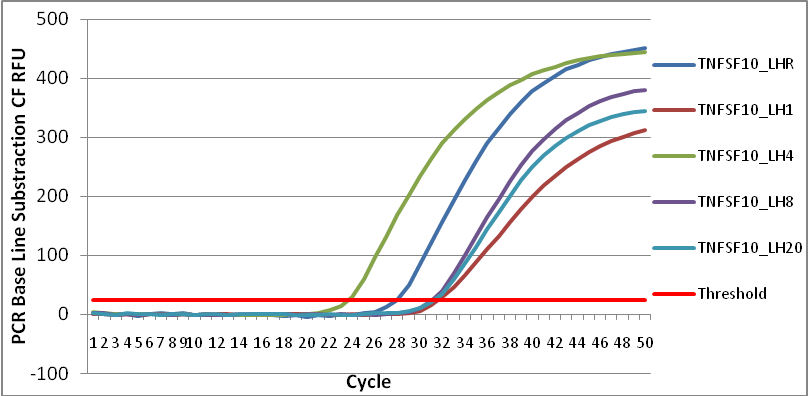
**

**B:**

**
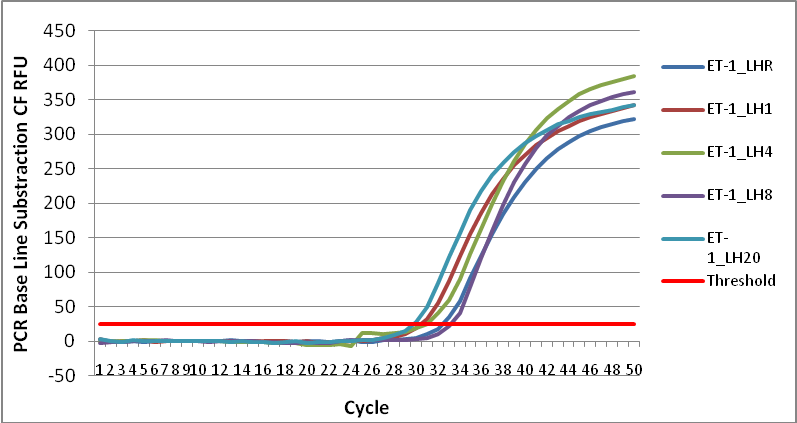
**
